# Ligand-based drug design of quinazolin-4(3H)-ones as breast cancer inhibitors using QSAR modeling, molecular docking, and pharmacological profiling

**DOI:** 10.1186/s43046-023-00182-3

**Published:** 2023-08-07

**Authors:** Sagiru Hamza Abdullahi, Adamu Uzairu, Gideon Adamu Shallangwa, Sani Uba, Abdullahi Bello Umar

**Affiliations:** Department of Chemistry, Faculty of Physical Sciences, Ahmadu Bello University, Zaria, Kaduna State, P.M.B.1045 Nigeria

**Keywords:** QSAR, Breast cancer, Ligand-based design, Molecular docking, Quinazolin-4(3H) derivatives

## Abstract

**Background:**

Breast cancer is the most common tumor among females globally. Its prevalence is growing around the world, and it is alleged to be the leading cause of cancer death. Approved anti-breast cancer drugs display several side effects and resistance during the early treatment stage. Hence, there is a need for the development of more effective and safer drugs. This research was aimed at designing more potent quinazolin-4(3H)-one molecules as breast cancer inhibitors using a ligand-based design approach, studying their modes of interaction with the target enzyme using molecular docking simulation, and predicting their pharmacological properties.

**Methods:**

The QSAR model was developed using a series of quinazoline-4(3H)-one derivatives by utilizing Material Studio v8.0 software and validated both internally and externally. Applicability domain virtual screening was utilized in selecting the template molecule, which was structurally modified to design more potent molecules. The inhibitive capacities of the design molecules were predicted using the developed model. Furthermore, molecular docking was performed with the EGFR target active site residues, which were obtained from the protein data bank online server (PDB ID: 2ITO) using Molegro Virtual Docker (MVD) software. SwissADME and pkCSM online sites were utilized in predicting the pharmacological properties of the designed molecules.

**Results:**

Four QSAR models were generated, and the first model was selected due to its excellent internal and external statistical parameters as follows: R^2^ = 0.919, R^2^_adj_ = 0.898, Q^2^_cv_ = 0.819, and R^2^_pred_ = 0.7907. The robustness of the model was also confirmed by the result of the Y-scrambling test performed with cR^2^p = 0.7049. The selected model was employed to design seven molecules, with compound 4 (pIC_50_ = 5.18) adopted as the template. All the designed compounds exhibit better activities ranging from pIC_50_ = 5.43 to 5.91 compared to the template and Doruxybucin (pIC_50_ = 5.35). The results of molecular docking revealed better binding with the EGFR target compared with the template and Doruxybucin. The designed compounds exhibit encouraging therapeutic applicability, as evidenced by the findings of pharmacological property prediction.

**Conclusions:**

The designed derivatives could be utilized as novel anti-breast cancer agents.

## Background

Cancer is one of the leading causes of casualties in the world, and as a result, there is an urgent need to design new and effective remedies. Presently, chemotherapy combined with unique working mechanisms is one of the techniques that are being embraced for most cancer therapies [1]. Breast cancer is observed in humans and other mammals; it originates from the breast tissue, either from the milk ducts, known as ductal carcinomas, or from the lobules, which supply milk to the ducts, called lobular carcinomas [2]. The majority of human breast cancer cases are in women, even though it is also rarely observed in men [3]. Breast cancer is the most prominent tumor among females globally. Its prevalence is growing around the world, and it is claimed to be the leading cause of cancer death, according to the American Cancer Society. The projected new cases of breast cancer in the USA (2014) are 235,030; 232,670 of them are estimated to be female, and the other 2360 are male. Additionally, the estimated number of breast cancer deaths is 40,430 for the same year [4].

Quinazolines play a key role in the medicinal chemistry arena: they are of specific attention and are considered favorable skeletons in the exploration of new bioactive agents because of their pronounced pharmacological activities such as anti-inflammatory [5], antimicrobial [6], antihypertensive [7], anticonvulsant [8], cholinesterase inhibitors [9], anticancer [10], and anti-diabetic [11].

The primary aim of anti-cancer drugs is to neutralize cancer cells without inflicting unfavorable damage on other normal cells. Regrettably, currently existing anti-cancer drugs result in several side effects, such as endometrial cancer and drug resistance. Hence, there is a severe need to discover and design novel anticancer agents with improved tumor selectivity, safety, and proficiency [12]. Computational methods are frequently utilized in most modern drug discovery approaches. These methods are very precise, faster, and cost-effective. The computational approach to drug design is essentially divided into two approaches: the ligand-based (LB) and structure-based (SB) processes. Quantitative structure–activity relationship (QSAR) is one of the computational approaches for drug discovery [13]. The generation of a robust QSAR model for the prediction of biological activity of a compound prior to its synthesis is paramount because a successful QSAR model enables the establishment of relationships between the structural features and biological activity of any class of compounds, as well as providing researchers with a profound investigation of the lead molecules to be utilized for supplementary research [14]. Another important aspect in the area of drug discovery is molecular docking, which aids in understanding the nature of ligand-receptor interaction, predicting the binding capability of amino acid residues to specific groups on the target receptor, and disclosing the strength of the interaction [15, 16].

This research was aimed at designing more potent quinazolin-4(3H)-one molecules as breast cancer inhibitors using a ligand-based design approach, studying their modes of interaction with the target enzyme using molecular docking simulation, and predicting their pharmacological properties.

## Methods

### Data set retrieval, geometry optimization, and descriptor calculations

A series of compounds containing thirty-five (35) quinazolin-4-one derivatives were obtained from the literature [17, 18]. Their biological activities (IC50) in µM were represented by the –log IC50. 2D structures of the derivatives were sketched using Perkin-Elmer ChemDraw software and then transformed to 3D format using Spartan v14.0 software. Geometry optimization of the molecules was performed on the Spartan interface using Density Functional Theory (DFT) quantum mechanical calculations with B3LYP/ 631-G* basis set. Geometry optimization was performed to find the most stable and least energetic conformer of the molecules [16]. The geometrically optimized structure of the molecules was kept in a single folder in Spatial Document File (SDF) format and then transported to the PADEL descriptor toolkit for the computation of the essential molecular descriptors that are responsible for the anti-breast cancer activities of the molecules [19].

### Data pretreatment and division

The calculated molecular descriptors of the compounds in the Excel sheet were pretreated manually and then using data pretreatment software to reduce redundant and irrelevant descriptors. The pretreated data was then separated into training and test sets using the Kennard-Stone algorithm utilized in data division software. In this research work, the training set comprises 25 molecules, while the test set is made up of 10 molecules.

### QSAR model building and validation

The training set molecules were utilized in the QSAR model generation and internal validations, while the test set molecules were utilized in the model's external validation and predictive strength appraisal. The genetic function algorithm (GFA) combined with multi-linear regression (MLR) in Material Studio v8.0 was utilized for the feature selection of the relevant descriptors and model generation. GFA produces a population of models rather than a single model [20]. In QSAR modeling, MLR correlates the independent variables (selected descriptors) with the dependent variables (experimental pIC50) [21]. The best QSAR model was identified and selected based on the following statistical metrics: correlation coefficient of the training set (R2training), adjusted correlation coefficient (R2adj), cross-validation coefficient (Q2cv), and correlation coefficient of the external test set (R2ext). The equations that defined the validation parameters are presented in Eqs. (1, 2, 3, and 4), respectively.

1 $${\mathrm{R}}^{2} = 1-\frac{{\sum ({Y}_{exp}-{Y}_{pred})}^{2}}{\sum ({Y}_{exp}-{{Y}_{mtraining})}^{2}}$$

2 $${{\mathrm{R}}^{2}}_{\mathrm{adj}} = 1-\left(1-{\mathrm{R}}^{2}\right) \frac{N-1}{N-P-1} = \frac{\left(N-1\right){R}^{2}-q}{N-P+1}$$

3 $${{\mathrm{Q}}^{2}}_{\mathrm{cv}}\hspace{0.17em}=\hspace{0.17em}1-\frac{{\sum ({Y}_{pred}-{Y}_{exp})}^{2}}{\sum ({Y}_{exp}-{{Y}_{mtraining})}^{2}}$$

4 $${{\mathrm{R}}^{2}}_{\mathrm{pred}}\hspace{0.17em}=\hspace{0.17em}1-\frac{{\sum ({Y}_{pred}-{Y}_{exp})}^{2}}{\sum ({Y}_{exp}-{{Y}_{mtraining})}^{2}}$$

Where P is the independent variable’s number, N represents the size of the training set sample, and the experimental, predicted, and mean activities of the training set samples are represented by Yexp, Ypred, and Ymtraining.

### Y-scrambling test

The Y-scrambling test was performed to ascertain the robustness of a model and also to confirm that a model was not obtained by chance correlation. In performing the Y-scrambling test, the experimental activities were reshuffled with the descriptors kept unchanged, and new QSAR models were generated for several trials. The newly developed QSAR models were anticipated to have low Q2 and R2 values. The statistical parameter for the Y-scrambling test is the coefficient of determination for the Y-scrambling test, cR2p (Eq. 5), and for a model to be reliable, its value must be greater than 0.5 [22].

5 $${\mathrm{cR}}^{2}\mathrm{p }=\mathrm{ R }\times {\left[{\mathrm{R}}^{2}-{R}_{r}^{2} \right].}^{2}$$

cR^2^p is coefficient of determination for Y-scrambling, *R* is the coefficient of determination for *Y*-randomization and *R*r is average ‘*R*’ of random models.

### Applicability domain (AD)

The hypothetical space within a chemical boundary that comprises the model descriptors and the modeled response is referred to as the applicability domain (AD) of a QSAR model. It enables the extrapolation of uncertainty in the detection of certain molecules based on the data set of molecules utilized in the model generation. The applicability domain aids in the detection of X-outliers from the training set and also discovers compounds that are outside the prescribed AD space [16]. Among the numerous approaches used to define the applicability domain of a robust QSAR model is the leverage approach [16]. The leverages of each molecule in the data set are utilized in this approach, which permits the investigation of the position of a new molecule in the QSAR model. The leverage value of each compound is calculated using the below equation.

6 $${h}_{i}={y}_{i} {\left({Y}^{T} Y\right)}^{-1}{{y }^{T}}_{i}$$

Where y indicates the vector descriptor of the referred sample and Y signifies the matrix of the descriptor obtained from the training set descriptor values. The threshold leverage (h*) was computed using Eq. 7 below:

7 $${h }^{*} = \frac{3(Q+1}{M}$$

M is the number of training set molecules, and Q is the number of independent variables (descriptors) used in developing the model. A plot of standardized residuals against the leverage values (h) of the compounds is called the William’s plot. When the leverage of a compound exceeds the threshold value (h*) and its residual value lies beyond the ± 3 defined space, it is presumed to have influenced the performance of the model, and the compound may be eliminated from the domain [22]. Hence, leverage and standardized residuals were jointly used to characterize and determine the applicability domain.

### Docking studies

Ligand–protein molecular docking studies were performed using Molegro Virtual Docker (MVD) software, as it yields better and more precise results compared to other docking software. All the ligands were prepared by optimization using DFT with the B3LYP/631G* basis set and then saved in the protein data bank (PDB) format. The EGFR target was retrieved from the protein data bank online site (http://www.rcsb.org/pdb/) with PDB ID: 2ITO and then prepared on the MVD work space by eliminating excess water molecules and the co-crystallized ligand enveloped in the crystal structure. The docking results were determined based on the set scoring functions, such as the MolDock score and Re-rank score [23, 24]. Visualization of various intermolecular interactions was performed by utilizing Discovery Studio v3.5 software.

### ADMET and drug-likeness prediction

The ADMET properties of a compound are crucial in determining its therapeutic effectiveness [25, 26]. SwissADME and pkCSM online tools were employed to assess the physicochemical properties, pharmacokinetics, and drug-like properties by utilizing the famous Lipinski’s rule of five.

## Results

### Result of QSAR studies

Four (4) QSAR models were developed using Material Studio v8.0 to offer an efficient prediction of the anti-breast cancer activities of the studied molecules. The mathematical expressions of the generated models are shown below:

#### Model 1

$${\mathrm{pIC}}_{50}\hspace{0.17em}=-0.145346037*\mathrm{ATSC}5\mathrm{p}\hspace{0.17em}+\hspace{0.17em}1.506479874*\mathrm{GATS}7\mathrm{e}-0.033543143*\mathrm{VR}{2}_{\mathrm{Dzs}}\hspace{0.17em}+\hspace{0.17em}0.369945024*\mathrm{ZMIC}3-0.334069472*\mathrm{ZMIC}4\hspace{0.17em}+\hspace{0.17em}2.221651$$

#### Model 2

$${\mathrm{pIC}}_{50}\hspace{0.17em}=-0.157468042*\mathrm{ATSC}5p\hspace{0.17em}+\hspace{0.17em}1.874776212*\mathrm{GATS}7\mathrm{e}-0.053075390*\mathrm{VR}2\_\mathrm{Dzs}\hspace{0.17em}+\hspace{0.17em}0.262551901*\mathrm{ZMIC}3-0.232183036*\mathrm{ZMIC}5\hspace{0.17em}+\hspace{0.17em}2.475692$$

#### Model 3

$${\mathrm{pIC}}_{50}\hspace{0.17em}=-0.143572361*\mathrm{ATSC}5\mathrm{p}\hspace{0.17em}+\hspace{0.17em}1.644069923*\mathrm{GATS}7\mathrm{e}-0.036310109*\mathrm{ZMIC}2\hspace{0.17em}+\hspace{0.17em}0.416554236*\mathrm{ZMIC}3-0.330830121*\mathrm{ZMIC}4\hspace{0.17em}+\hspace{0.17em}1.713922$$

#### Model 4

$${\mathrm{pIC}}_{50}\hspace{0.17em}=-0.146348290 *\mathrm{ ATSC}5\mathrm{p}\hspace{0.17em}+\hspace{0.17em}1.596869177 *\mathrm{ GATS}7\mathrm{e}-0.000590607 *\mathrm{ VR}1\_\mathrm{Dzs}\hspace{0.17em}+\hspace{0.17em}0.359099432 *\mathrm{ ZMIC}3-0.314703957 *\mathrm{ ZMIC}4\hspace{0.17em}+\hspace{0.17em}1.744154$$

### Results of internal and external validations of the developed models

**Fig. 1:**
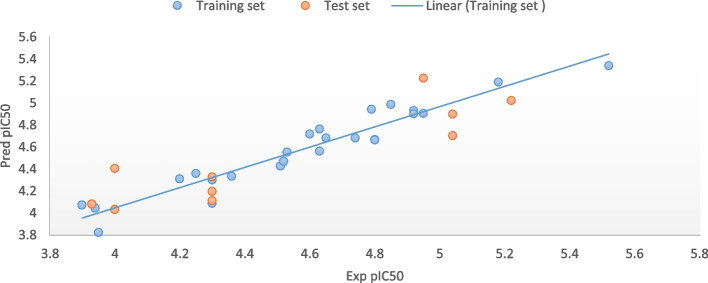
Plot of experimental pIC_50_ of the training and test sets against their predicted activities

**Fig. 2:**
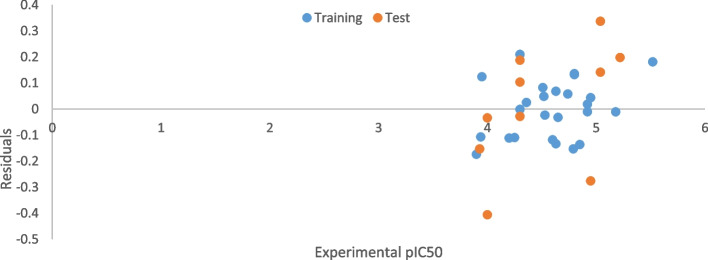
Plot of experimental activities of the training and test set against their residuals

**Fig. 3:**
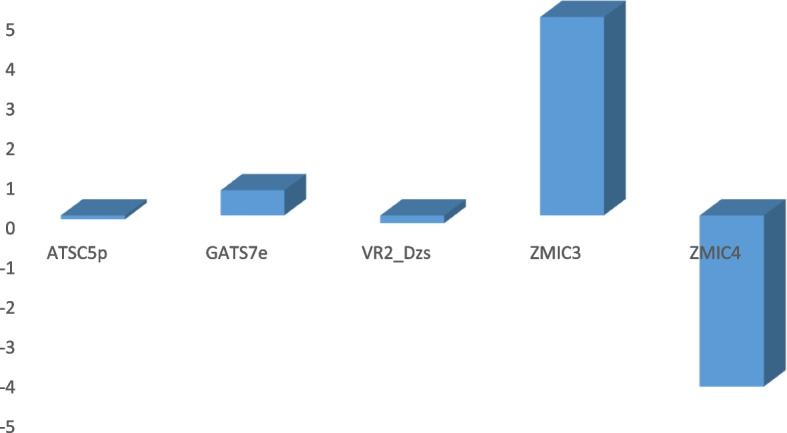
Mean effect values of the relevant descriptors

The results of internal and external validations of the developed QSAR models are presented in Tables 1 and 2 and were found to have passed the minimum requirements of an acceptable QSAR model [27]. However, model 1 with the best internal validation metrics (Table 1) was selected for the external validation studies using the test set molecules. The activities of the test set molecules were predicted using the model, and Eq. 4 was utilized to compute the predicted correlation coefficient (R^2^_pred_) in order to access the selected model's predictive capacity. For the sake of replication, the descriptor values, the experimental and predicted activities of the test set molecules, and the step-by-step calculations of the external prediction correlation coefficient (R^2^_pred_) are presented in Table 2. The structure of the quinazoline-4-one molecules, their experimental and predicted activities are presented in Table 3. The experimental activities (Figs. 1 and 2) were plotted against the predicted activities as presented in Fig. 3, while the plot of experimental activities against the standardized residuals of the molecules are presented in Fig. 5.

**Table 1:** Minimum acceptable values of the developed QSAR models

Symbol	Definition	Threshold value	Model 1	Model 2	Model 3	Model 4
R^2^	Correlation coefficient of the training set	≥ 0.6	0.919473	0.904997	0.9049	0.903554
R^2^_adj_	Adjusted R^2^	≥ 0.6	0.898281	0.879996	0.879874	0.878174
Q^2^_CV_	Cross validation coefficient	≥ 0.5	0.819201	0.819222	0.787238	0.799009
R^2^—Q^2^_cv_	Difference between R^2^ and Q^2^_cv_	≤ 0.3	0.10072	0.085755	0.117662	0.104545
P(95%)	Confidence interval at 95% confidence level	< 0.05	0.097933	0.106372	0.106426	0.107177

**Table 2:** External validation of the selected model

ID	ATSC5p	GATS7e	VR2_Dzs	ZMIC3	ZMIC4	Y_exp_	Y_pred_	(Y_exp_ – Y_pred_)	(Y_exp_ – Y_mtrng_)	(Y_exp_ – Y_pred_)^2^	(Y_exp_ – Y_mtrng_)^2^
10	3.158387	0.832666	13.67159	27.5155	25.91358	3.93	4.08	-0.150	-0.660	0.020	0.435
16	-1.72771	0.983945	22.10245	38.34185	36.4481	4.95	5.23	-0.280	0.360	0.080	0.129
18	0.415765	1.010939	13.98274	37.49586	36.12086	5.22	5.02	0.190	0.630	0.040	0.397
19	-1.61582	0.867801	14.44765	36.7727	35.8813	5.04	4.89	0.140	0.450	0.020	0.203
25	4.038224	1.120313	11.92254	38.35934	37.15472	5.04	4.70	0.340	0.450	0.110	0.203
30	-1.69198	0.400794	14.24406	29.02303	27.59978	4.30	4.11	0.190	-0.290	0.030	0.084
32	-2.67056	0.354307	19.20165	30.28259	28.95225	4.00	4.03	-0.030	-0.590	0.001	0.348
35	-2.74816	0.40653	14.33373	30.44555	29.00592	4.30	4.33	-0.030	-0.290	0.001	0.084
36	-3.1579	0.379647	13.6011	31.80867	30.41389	4.00	4.41	-0.410	-0.590	0.165	0.348
37	-0.91779	0.490773	13.44224	30.23823	28.84345	4.30	4.19	0.100	-0.290	0.011	0.084
									Sum	0.484	2.315
									${{\mathrm{R}}^{2}}_{\mathrm{pred}}=1-\frac{{\sum (\mathrm{Yexp}-\mathrm{Ypred })}^{2}}{{\sum (\mathrm{Yexp}-\mathrm{Ymtrng })}^{2}}$		$=1-\frac{0.484}{2.315}$
											$${{\mathrm{R}}^{2}}_{\mathrm{pred}}=0.791$$

**Table 3:** Structures, experimental, predicted activities and residuals of Quinazolin-4-one derivatives against MCF-7 breast cancer cell line

S/NO	STRUCTURE	Exp pIC_50_	Pred Pic_50_	Residual
1		3.90	4.07	-0.17
2		3.95	3.83	0.12
3		4.80	4.67	0.13
4		5.18	5.19	-0.01
5		4.25	4.36	-0.11
6		4.36	4.34	0.02
7		4.53	4.55	-0.02
8		4.51	4.43	0.08
9^a^		3.93	4.08	-0.15
10		3.94	4.05	-0.11
11		4.60	4.72	-0.12
12		4.80	4.66	0.14
13		4.52	4.47	0.05
14		4.20	4.31	-0.11
15^a^		4.95	5.23	-0.28
16		4.79	4.94	-0.15
17^a^		5.22	5.02	0.20
18^a^		5.04	4.89	0.15
19		5.52	5.34	0.18
20		4.92	4.93	-0.01
21		4.92	4.90	0.02
22		4.63	4.56	0.07
23		4.85	4.99	-0.14
24^a^		5.04	4.70	0.34
25		4.95	4.91	0.04
26		4.74	4.68	0.06
27		4.63	4.76	-0.05
28		4.65	4.68	-0.03
29^a^		4.30	4.11	0.19
30		4.30	4.09	0.21
31^a^		4.00	4.03	-0.03
32		4.30	4.30	0.00
33^a^		4.30	4.33	-0.03
34^a^		4.00	4.41	-0.41
35^a^		4.30	4.19	0.11
	DOROXUBUCIN	5.35		

^a^Test set compounds

### Result of Y-scrambling test

The results of Y-scrambling test, performed to ascertain the robustness of a QSAR model as well as ensuring that the model was not obtained accidentally was presented in Table 4.

**Table 4:** Result of Y-scrambling test for the selected model

Model	R	R^2^	Q^2^
Original	0.891074	0.794014	0.66778
Random 1	0.52001	0.270411	-0.3075
Random 2	0.301356	0.090816	-0.32506
Random 3	0.434349	0.188659	-0.4247
Random 4	0.361105	0.130397	-0.34535
Random 5	0.149213	0.022265	-0.50583
Random 6	0.555151	0.308193	-0.03051
Random 7	0.308871	0.095401	-0.33896
Random 8	0.475642	0.226235	-0.10752
Random 9	0.530334	0.281254	-0.11649
Random 10	0.465513	0.216702	-0.59389
Random Models Parameters			
Average r:	0.410154		
Average r^2^:	0.183033		
Average Q^2^:	-0.30958		
cRp^2^:	0.7049		

### Results of descriptor mean effect and William’s plot of the selected model

**Fig. 4:**
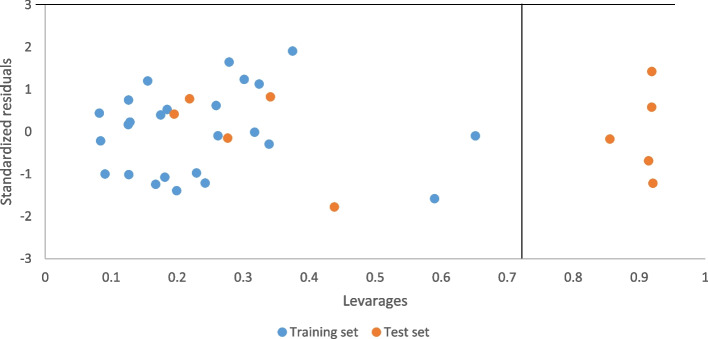
William’s plot of the selected model

The result of the model descriptors mean effect values is presented in a chart format in Fig. 3, while the model William’s plot is presented in Fig. 4.

### Ligand-based drug design

**Fig. 5:**
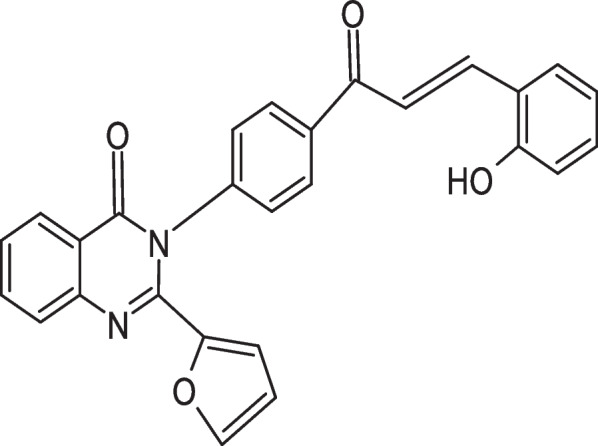
Structure of compound 4

**Fig. 6:**
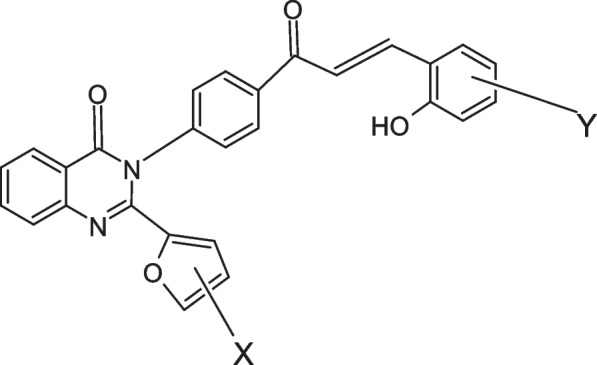
Structure of the template used for the design

The structures of compound 4 and the template utilized for the ligand-based design process are presented in Figs. 5, and 6. Seven compounds were designed via the structural adjustment of the template by the addition of active groups and fragments at the specified X, and Y positions. The inhibitive capacities of the molecules were predicted using the selected model. 2D structures and the predicted activities (pIC_50_) of the designed molecules were presented in Table 5.

**Table 5:** Structure and pIC_50_ of the designed compounds

S/no	Structure	pIC_50_
1		5.69
2		5.91
3		5.76
4		5.43
5		5.59
6		5.63
7		5.49

### Results of docking studies

The docking scores of the designed molecules, and residual interactions are presented in Table 6, while Figs. 9 - 15 represent the 2D and 3D interactions of the designed molecules with the EGFR target active site residues.

**Table 6:** Docking scores and several Amino acid residues interactions between the designed compounds and the active sites of the EGFR receptor (pdb id = 2ITO)

S/no	MolDock score	Rerank score	amino acid residues	Category	Type of interactions
1	-137.652	-100.296	LYS745 ARG841 ARG841 ASP837 VAL726 LYS745 PRO877 LYS745 LEU747 ILE759 ALA722	Electrostatic Electrostatic Electrostatic Electrostatic Hydrophobic Hydrophobic Hydrophobic Hydrophobic Hydrophobic Hydrophobic Hydrophobic	Pi-Cation Pi-Cation Pi-Cation Pi-Anion Alkyl Alkyl Pi-Alkyl Pi-Alkyl Pi-Alkyl Pi-Alkyl Pi-Alkyl
2	-157.482	-119.221	GLY857 GLY857 GLU762 ARG841 GLU758 ASP837 PHE723 ALA755 LEU747 ILE759 PRO877	Hydrogen bond Hydrogen bond Hydrogen bond Electrostatic Electrostatic Electrostatic Hydrophobic Hydrophobic Hydrophobic Hydrophobic Hydrophobic	Conventional Carbon-Hydrogen Carbon-Hydrogen Pi-Cation Pi-anion Pi-anion Pi-Pi stacked Pi-alkyl Pi-alkyl Pi-alkyl Pi-alkyl
3	-153.96	-105.477	THR854 THR790 GLY796 ASP800 LYS745 MET766 MET766 CYS797 LYS745 MET766 LEU788 LEU718 LYS728 LEU792 VAL726 VAL726 ALA743 LYS745	Hydrogen bond Hydrogen bond Hydrogen bond Hydrogen bond Electrostatic Other Other Other Hydrophobic Hydrophobic Hydrophobic Hydrophobic Hydrophobic Hydrophobic Hydrophobic Hydrophobic Hydrophobic Hydrophobic	Conventional Carbon-Hydrogen Carbon-Hydrogen Carbon-Hydrogen Pi-Cation Pi-Sulfur Pi-Sulfur Pi-Sulfur Alkyl Alkyl Alkyl Pi-Alkyl Pi-Alkyl Pi-Alkyl Pi-Alkyl Pi-Alkyl Pi-Alkyl Pi-Alkyl
4	-156.961	-53.2419	LYS745 MET793 CYS775 MET793 LEU844 VAL726 LYS745 LEU718 ALA743 MET793 LEU844	Hydrogen bond Hydrogen bond Hydrophobic Hydrophobic Hydrophobic Hydrophobic Hydrophobic Hydrophobic Hydrophobic Hydrophobic Hydrophobic	Conventional Pi-Donor Alkyl Alkyl Alkyl Pi-Alkyl Pi-Alkyl Pi-Alkyl Pi-Alkyl Pi-Alkyl Pi-Alkyl
5	-150.758	-84.7712	LYS745 SER719 ASP800 ASP855 LEU718 LEU718 VAL726 VAL726 ALA743 LYS745 MET766 LEU788 LEU844 CYS797 LEU718	Hydrogen bond Hydrogen bond Electrostatic Electrostatic Hydrophobic Hydrophobic Hydrophobic Hydrophobic Hydrophobic Hydrophobic Hydrophobic Hydrophobic Hydrophobic Hydrophobic Hydrophobic	Conventional Carbon-Hydrogen Pi-Anion Pi-Anion Pi-Sigma Alkyl Alkyl Pi-Alkyl Pi-Alkyl Pi-Alkyl Pi-Alkyl Pi-Alkyl Pi-Alkyl Pi-Alkyl Pi-Alkyl
6	-161.369	-117.521	GLY724 LYS745 GLU762 ASP855 PHE723 PHE723 ALA755 LEU747 ALA743 LEU844 LEU718 LEU792 VAL726 LYS745 LEU747 ILE759	Hydrogen bond Hydrogen bond Electrostatic Electrostatic Hydrophobic Hydrophobic Hydrophobic Hydrophobic Hydrophobic Hydrophobic Hydrophobic Hydrophobic Hydrophobic Hydrophobic Hydrophobic Hydrophobic	Conventional Pi-Cation Pi-Anion Pi-Anion Pi-Sigma Pi-Pi T-shaped Alkyl Alkyl Pi-Alkyl Pi-Alkyl Pi-Alkyl Pi-Alkyl Pi-Alkyl Pi-Alkyl Pi-Alkyl Pi-Alkyl
7	-162.572	-127.635	GLY724 ALA755 LYS745 GLU762 PHE723 PHE723 ALA743 LEU844 LEU718 ALA743 VAL726 LYS745 VAL726 LEU747 ILE759	Hydrogen bond Hydrogen bond Hydrogen bond Electrostatic Hydrophobic Hydrophobic Hydrophobic Hydrophobic Hydrophobic Hydrophobic Hydrophobic Hydrophobic Hydrophobic Hydrophobic Hydrophobic	Conventional Conventional Pi-Cation Pi-Anion Pi-Sigma Pi-Pi T-shaped Pi-Alkyl Pi-Alkyl Pi-Alkyl Pi-Alkyl Pi-Alkyl Pi-Alkyl Pi-Alkyl Pi-Alkyl Pi-Alkyl
Template	-133.711	-103.969	LYS745 ASN842 ARG841 ASP855 ARG841 VAL726 ALA743 LYS745 LEU788 LEU844 ARG841 LEU718 VAL726	Hydrogen bond Hydrogen bond Hydrogen bond Electrostatic Hydrophobic Hydrophobic Hydrophobic Hydrophobic Hydrophobic Hydrophobic Hydrophobic Hydrophobic Hydrophobic	Conventional Conventional Pi-Cation Pi-Anion Pi-Sigma Pi-Alkyl Pi-Alkyl Pi-Alkyl Pi-Alkyl Pi-Alkyl Pi-Alkyl Pi-Alkyl Pi-Alkyl
DOX	-104.364	-29.958	ASP855 PHE723 ALA755 GLU762 ASP837 ASP855 GLY724 LYS745 LYS745 GLU762 PHE723 ALA722 LYS745	Hydrogen bond Hydrogen bond Hydrogen bond Hydrogen bond Hydrogen bond Hydrogen bond Hydrogen bond Electrostatic Hydrogen bond Electrostatic Hydrophobic Hydrophobic Hydrophobic	Conventional Conventional Conventional Conventional Conventional Conventional Carbon-Hydrogen Pi-Cation Pi-Cation Pi-Anion Pi-Pi T-shaped Pi-Alkyl Pi-Alkyl

### Results of pharmacokinetics and ADMET properties studies

Table 7 presents the results of drug-like check of the designed compounds by utilizing the famous rule of five proposed by Lipinski and co-workers, which was obtained using the SwissADME online server [28, 29]. While Table 8 presents the results of ADMET profiling obtained using the pkCSM online site.

**Table 7:** Pharmacokinetic properties of the designed compounds

S/no	MW	HBA	HBD	mlogP	TPSA	ABS SCORE	SA	Lipinski violation
1	518.95	5	1	4.01	85.33	0.55	3.81	1
2	500.5	6	1	2.48	98.47	0.55	3.91	1
3	518.95	5	1	3.74	85.33	0.55	3.74	1
4	518.95	5	1	4.01	85.33	0.55	3.82	1
5	518.95	5	1	4.01	85.33	0.55	3.83	1
6	534.95	6	1	2.94	98.47	0.55	3.92	1
7	515.52	6	2	1.96	124.49	0.55	4.06	1

*MW* Molecular Weight, *HBA* Hydrogen Bond Acceptors, *HBD* Hydrogen Bond Donors, *TPSA* Topological Polar Surface Area, *SA* Synthetic Accessibility

**Table 8:** Predicted ADMET properties of the designed compounds

S/no	Absorption Intestinal (Human) Absorption	Distribution LogBB LogPS		Metabolism Substrate Inhibitors 2D6 3A4 1A2 2C19 2C9 2D6 3A4							Excretion Total clearance	Toxicity AMES
1	97.183	-0.976	-1.532	NO	YES	YES	NO	NO	NO	YES	0.355	NO
2	100	-1.007	-1.809	NO	YES	YES	YES	YES	NO	YES	0.715	NO
3	98.517	-0.970	-1.353	NO	YES	NO	NO	NO	NO	YES	0.594	NO
4	96.265	-0.992	-1.577	NO	YES	YES	NO	NO	NO	YES	0.714	NO
5	97.718	-0.991	-1.529	NO	YES	YES	NO	NO	NO	YES	0.389	NO
6	100	-1.195	-1.698	NO	YES	NO	YES	YES	NO	YES	0.840	NO
7	100	-1.142	-1.948	NO	YES	NO	YES	YES	NO	YES	0.542	NO

## Discussion

The four developed QSAR models passed the minimum requirement for an acceptable model, as illustrated by their statistical parameters (Table 1). Model 1 was selected as the most relevant model as it has the best statistical significance. Its internal R^2^ value close to unity is an indicator that the selected model clarified an excessive proportion of the independent variable (molecular descriptor), sufficiently enough for a powerful QSAR model. A value of 0.919 suggests that 91.9% of the disparity lies in the residual, suggesting a very good model [23, 30]. Additionally, adjusted R^2^ has a very high value that is close to the internal R^2^ value for the selected model. This affirmed that the model possessed exceptional descriptive power for the response variables it contained and also illustrated the actual impact of the descriptors on the anti-cancer activities of the compounds. Additionally, to further confirm the robustness of the selected model, it was validated externally (Table 2), and the external validation correlation coefficient (R^2^_pred_) was found to be 0.791. This value exceeds the minimum recommended value of ≥ 0.6 for an acceptable model [16]. A high external prediction correlation coefficient (R^2^_pred_) indicates that the model can effectively predict the activities of new molecules. Hence, we can confidently conclude that the selected model will predict the anti-breast cancer activity of the quinazolin-4-one molecules accurately. Moreover, the selected model was utilized to predict the activity of both the training and test sets; the result is shown in Table 3. The experimental pIC_50_ of both the training and test sets were plotted against their predicted activities (Fig. 1), and a plot of experimental activities for the MCF-7 cell line against their residuals was presented in Fig. 2.

### Y-scrambling test

The result of the Y-scrambling test is shown in Table 4. The coefficient of determination for the Y-scrambling test cR^2^p was found to be 0.7049 for this test, which suggested that the model was not obtained by chance correlation and that it is powerful enough for the prediction of anti-breast cancer activities of molecules [20].

### Mean effect

The impact and contribution of each descriptor in a QSAR model were measured by computing its mean effect value (MF) [25]. The mean effect values of the selected descriptors are depicted in Fig. 3, respectively. The magnitude and signal of a descriptor are related to the biological activity of a compound [16]. A descriptor with a negative sign illustrates that the biological activity of a compound decreases by increasing its value, while a positive signal suggests that biological activity increases by increasing its value. In this study, the most important molecular descriptor is ZMIC3, a 2D class descriptor defined as a Z-modified information content index (neighborhood symmetry of 3-order). It has a mean effect value of 4.975, which suggests that an increase in its value affects the anti-cancer activities of the compounds positively. The least important descriptor is ZMIC4, another 2D class descriptor Z-modified information content index (neighborhood symmetry of 4-order); it has a mean effect value of -4.309, suggesting that the anti-cancer activities of the compounds can only be affected positively when its value is decreased. Another important descriptor that appears in the model is GATS7e, which is a 2D class autocorrelation descriptor defined as Geary autocorrelation—lag 7/ weighted by Sanderson electro negativities. Its positive mean effect value (0.624) suggested that the increasing biological activity of the compounds is related to the rise in the value of this descriptor. The value of this descriptor is increased by introducing groups with electronegative atoms to the main scaffold structure of the compound. Other descriptors that appear in the model are ATSC5p and VR2_Dzs, their negative mean effect values suggest that they are negatively related to the biological activities of the compounds. A chart showing the mean effect values of the descriptors that appear in the model is shown in Fig. 3.

### William’s plot of the selected model

The Williams plot for the model selected is shown in Fig. 4. The threshold leverage was found to be 0.72, and as such, only five compounds from the test set data lie beyond the defined AD (i.e., having h > h*). These compounds are labeled as influentials since the model performance is affected by them, but they may not be regarded as structural outliers since their residual values lie within the ± 3 region, which covers up to 99% of the uniformly distributed data [25].

### Ligand-based drug design

An in-silico screening approach was used for the design of novel quinazolin-4-ones with pIC_50_ activities against the MCF-7 cell line based on the selected QSAR model. Compound 4 from the training set samples was selected as a template for the design due to its high inhibitory activity (pIC50 = 5.18) and low standardized residual value (-0.01), which are within the defined domain of applicability. The structure of compound 4 and the template used for the design are shown in Figs. 5 and 6, respectively. The adjustment of the compound was done so that its synthesis experimentally would be easy and feasible. Virtual screening was applied by the addition and replacement of several entities at X and Y positions, as shown in Figs. 5 and 6. Seven (7) new potent compounds with improved activities which ranged from pIC_50_ = 5.43 to 5.91 compared to the template and Doruxybucin (pIC_50_ = 5.35) were designed. The structures of the designed compounds and their predicted activities are shown in Table 5.

### Result of Molecular docking studies

**Fig. 7:**
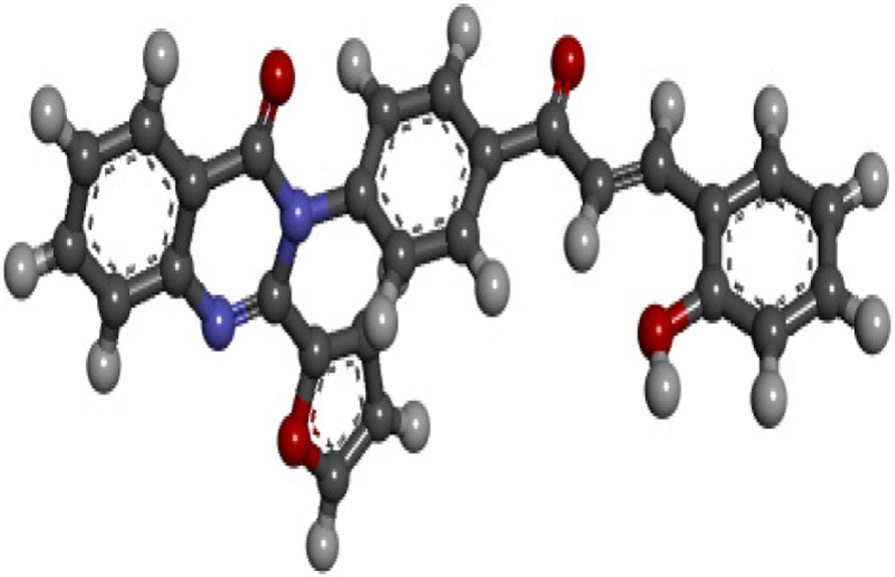
3D structure of the Template

**Fig. 8:**
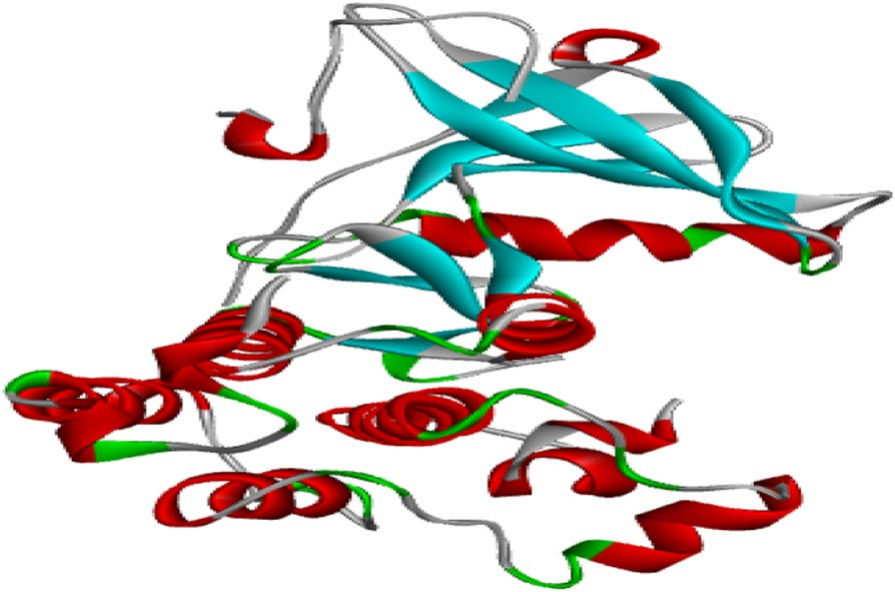
3D structure of the prepared EGFR receptor (pdb id = 2ITO)

The designed quinazoline-4-one derivatives were docked onto the active site of the EGFR receptor to explore the nature of interactions between the ligand and the target receptor. The template and the reference drug (DOX) were also docked on the same binding site to validate the docking studies. All the designed compounds were found to have better docking scores which ranged from -137.652 to -162.572 MolDock score and -53.2419 to -127.635 Re-rank score compared to the template (MolDock score = -133.711, Re-rank score = -103.969) and Doruxybucin (MolDock score = -104.364, Re-rank score = .-29.958). The higher binding affinities of the designed compounds disclosed that they binds more effectively with the EGFR target compared to Doruxybucin. The 3D structures of the template and prepared EGFR receptor are shown in Figures. 7 and 8, respectively, while the docking scores and various kinds of amino acid interactions between the designed quinazoline-4-ones and the active site of the EGFR receptor are presented in Table 6.

### Interpretation of the docking results

**Fig. 9:**
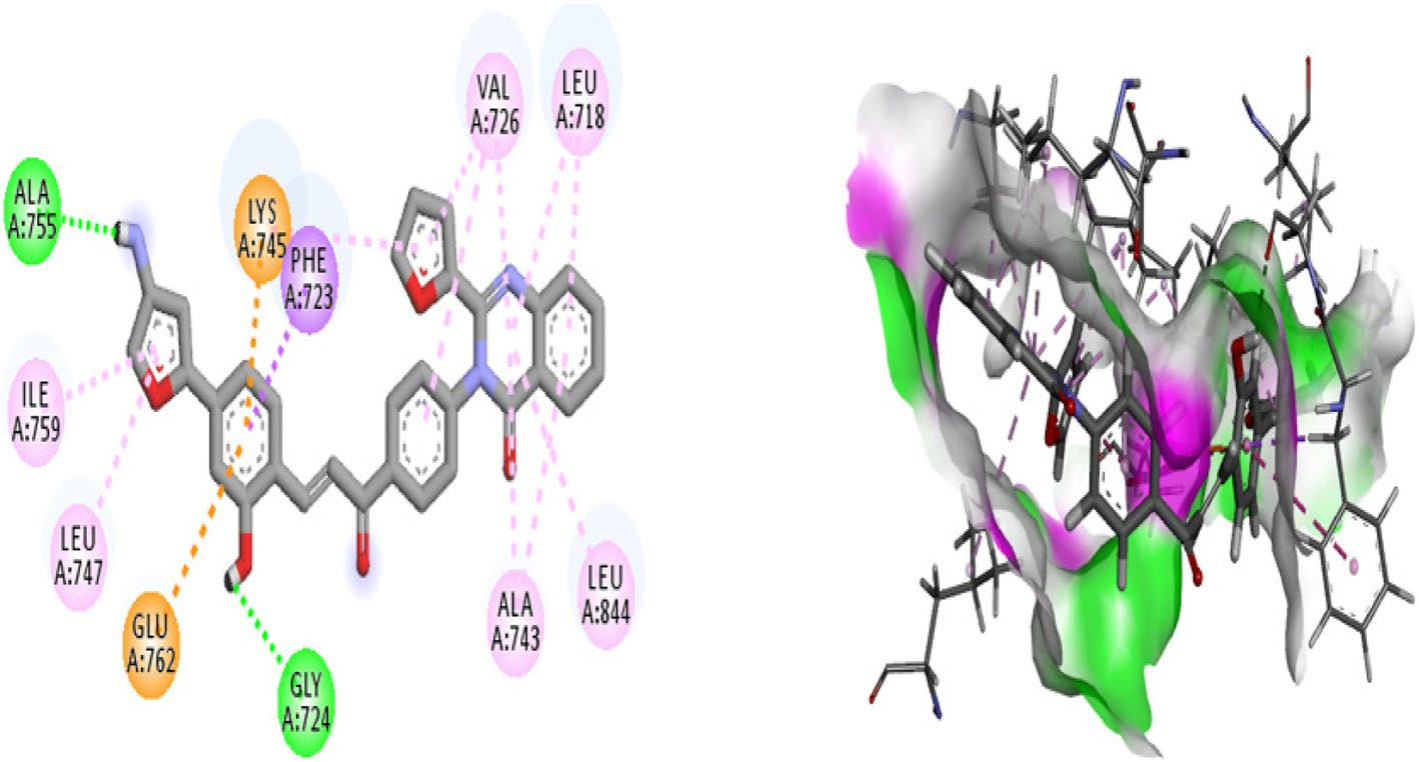
2D and 3D interactions of designed analogue 7 with the active site of the in the active site of the EGFR receptor

Designed analogue 7 had the best docking scores (MolDock score = -162.572, Rerank score = -127.635), and it is found to have interacted with the active site of the EGFR receptor through two (2) conventional hydrogen bonds, a single Pi-cationic hydrogen bond, a single electrostatic Pi-anion interaction, a hydrophobic Pi-Sigma and Pi-Pi T-shaped interaction, and several hydrophobic Pi-Alkyl interactions. GLY247 forms a conventional hydrogen bond with the hydrogen atom of the ortho-hydroxyl group attached to the benzene ring; another conventional hydrogen bond is between the amino group hydrogen atom attached to the furyl group. The β-position benzene ring is intercalated in space and forms a Pi-cationic hydrogen bond with LYS745 residue, an electrostatic Pi-anionic interaction with GLU762 residue, and hydrophobic Pi-Sigma and Pi-Pi T-shaped interactions with PHE723. LEU718, VAL726, ALA743, LEU844, LYS745, VAL726, LEU747, and ILE759 residues form pi-alkyl interactions with the ligand. 3D and 2D interactions of designed molecule 7 in the active site of the EGFR receptor are presented in Fig. 9.

**Fig. 10:**
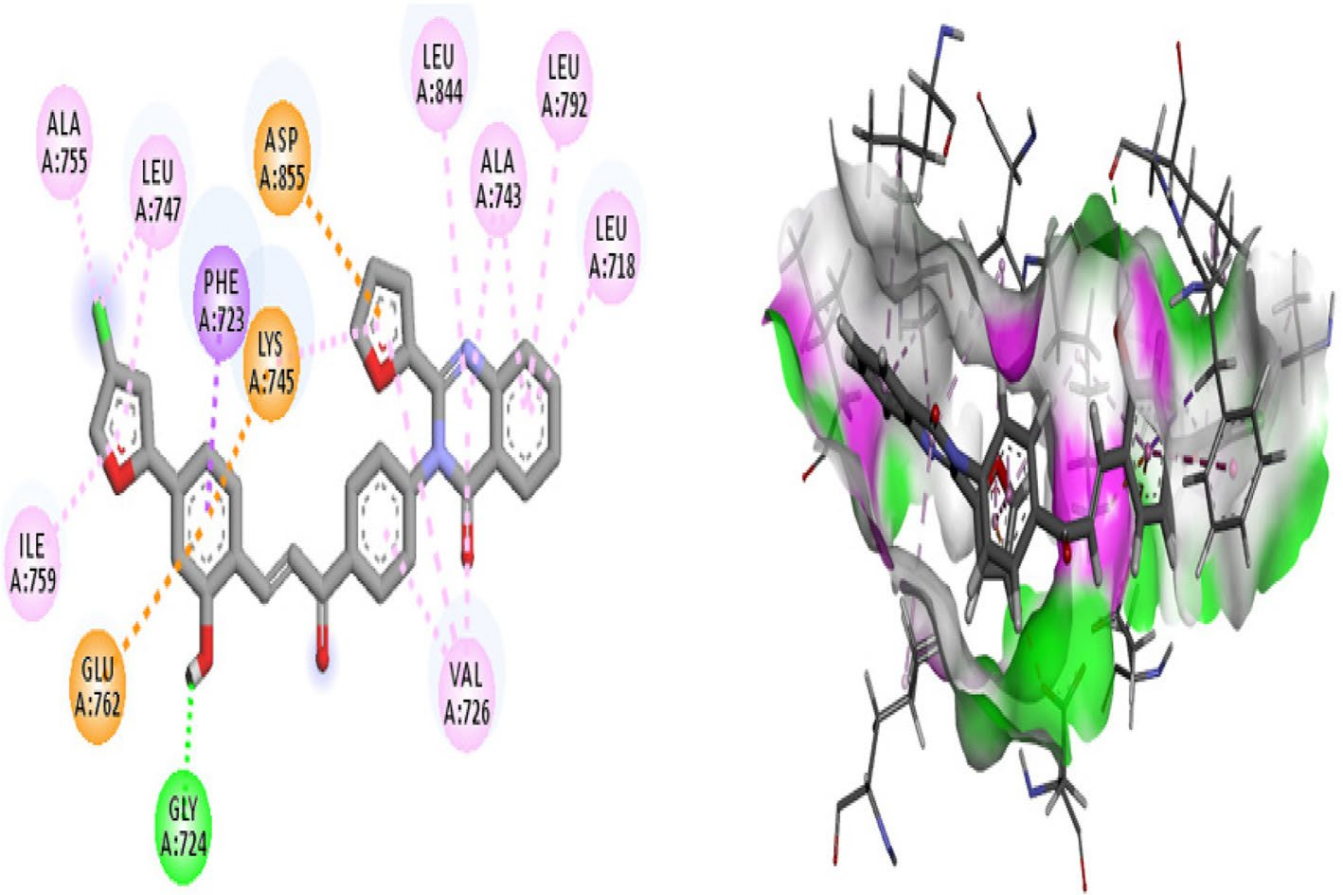
3D and 2D Interactions of designed compound 6 with the active site of the EGFR receptor

Designed compound 6 has the second-best docking score (MolDock score = -161.369, Rerank score = -117.521). It is observed to have interacted with the active site of the EGFR receptor via conventional and pi-cationic hydrogen bonds, two electrostatic pi-anion interactions, hydrophobic pi-sigma and pi-pi T-shaped interactions, two alkyl and several pi-alkyl interactions. GLY724 forms a conventional hydrogen bond with a hydrogen atom attached to the ortho-hydroxyl group of the β-Benzene ring, which is further intercalated in space to form a pi-cationic hydrogen bond with LYS745 and an electrostatic pi-anionic interaction with the GLU762 residue. Furyl ring moiety is intercalated in space and forms an electrostatic Pi-Anion interaction with ASP855. PHE723 forms hydrophobic Pi-sigma and Pi-Pi T-shaped interactions with the β-Benzene ring moiety. ALA755 and LEU747 form alkyl interactions with the chlorine atom attached to the furyl ring. ALA743, LEU844, LEU718, LEU792, VAL726, LYS745, LEU747, and ILE759 form pi-alkyl interactions with the compound. Fig. 10 represents the 2D and 3D interactions of designed compound 6 in the active site of the EGFR receptor.

**Fig. 11:**
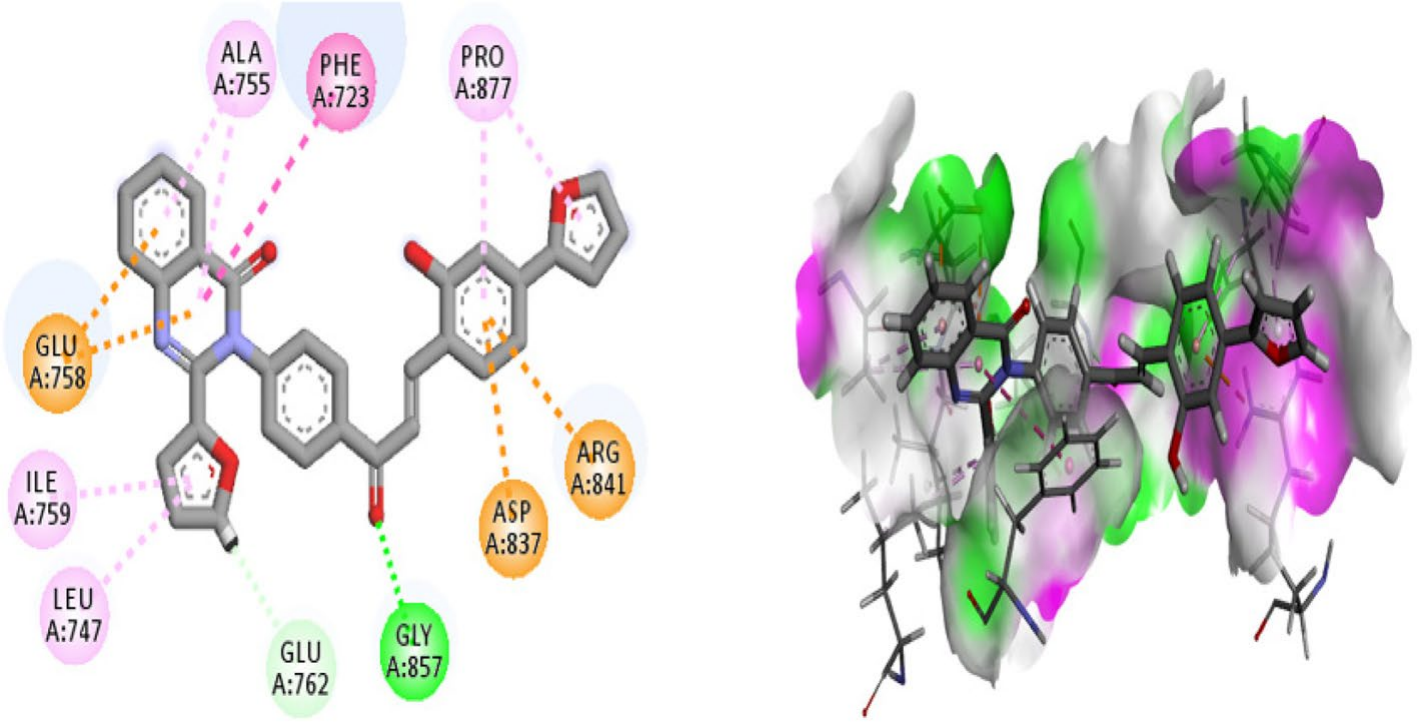
2D and 3D Interactions of designed compound 2 with the active site of the EGFR receptor

Designed compound 2 also has the third best docking score (MolDock score = -157.482 Rerank score = -119.221) and was found to interact with the active pocket of the receptor via a conventional hydrogen bond, two carbon-hydrogen bonds, electrostatic Pi-cation and Pi-anion interactions, hydrophobic Pi-Pi stacked interactions, and Pi-alkyl interactions. Carbonyl oxygen attached to the phenyl ring forms conventional and carbon-hydrogen bonds with GLY857; other carbon-hydrogen bonds are formed between the hydrogen atom (H17) of the furyl ring and GLU762. β-Benzene ring intercalated in space and forms an electrostatic Pi-cation interaction with ARG841 and a Pi-anion interaction with ASP837; other electrostatic Pi-anion interactions are between GLU758 and quinazoline scaffold. PHE723 forms a hydrophobic Pi-Pi stack, while ALA755, LEU747, ILE759, and PRO877 form Pi-Alkyl interactions. 3D and 2D interactions of compound 2 with the active sites of the EGFR receptor are shown in Fig. 11, respectively.

**Fig.12:**
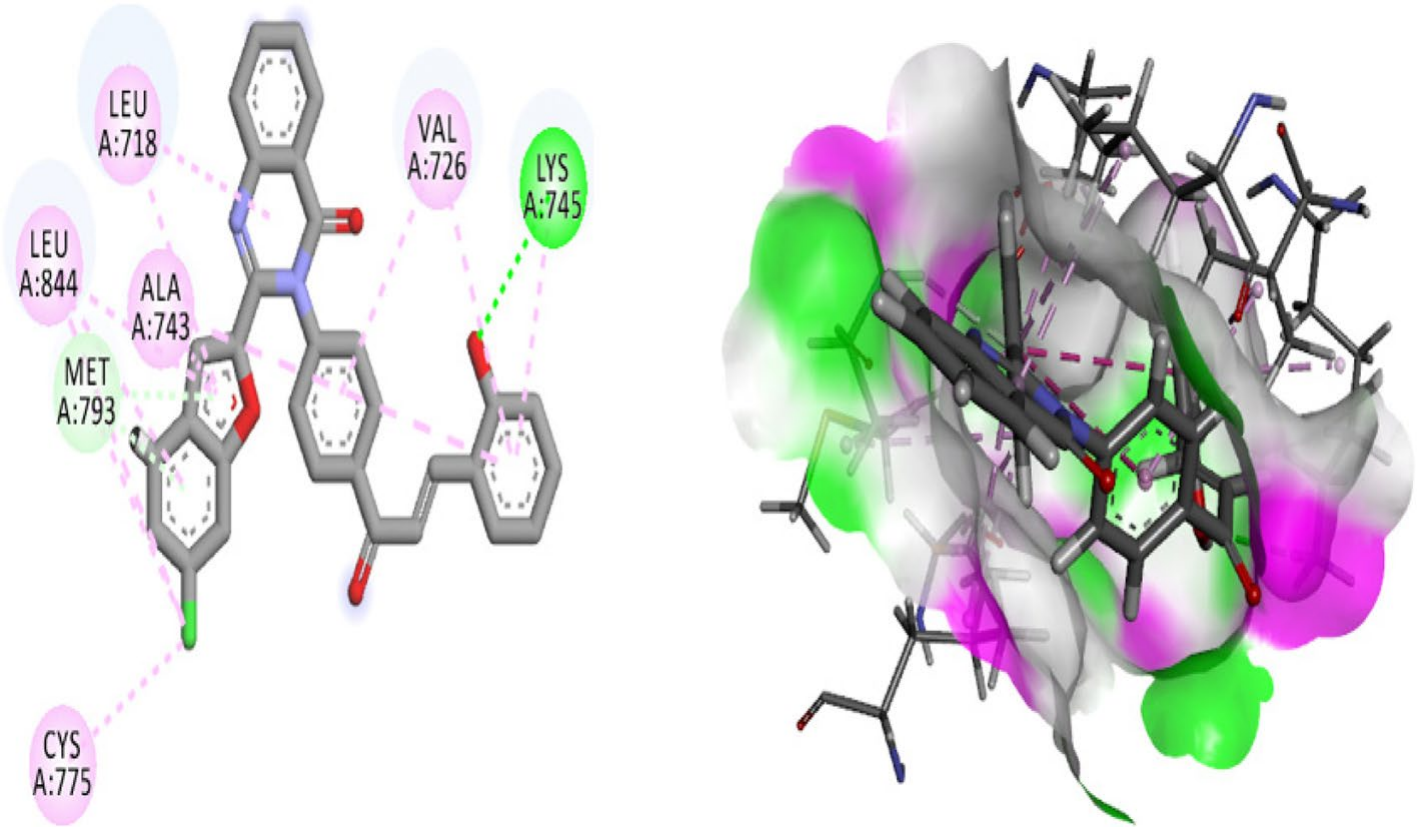
2D and 3D Interactions of designed compound 4 with the active site of the EGFR receptor

Designed compound 4 also has promising docking scores (MolDock score = -156.961, Rerank score = -53.2419). It is found to interact with the active site of the EGFR receptor via a single conventional hydrogen bond, two carbon-hydrogen bonds, three hydrophobic alkyls, and several pi-alkyl interactions. The oxygen atom of the ortho-hydroxyl group attached to the β-phenyl ring forms a conventional hydrogen bond with LYS745. MET793 forms double Pi-donor hydrogen bonds with the quinazoline scaffold. CYS775, MET793, and LEU844 form alkyl interactions, while VAL726, LYS745, LEU718, ALA743, MET793, and LEU844 residues form hydrophobic pi-alkyl interactions. Fig. 12 shows the 2D and 3D interactions of designed compound 4 in the active site of the EGFR receptor.

**Fig. 13:**
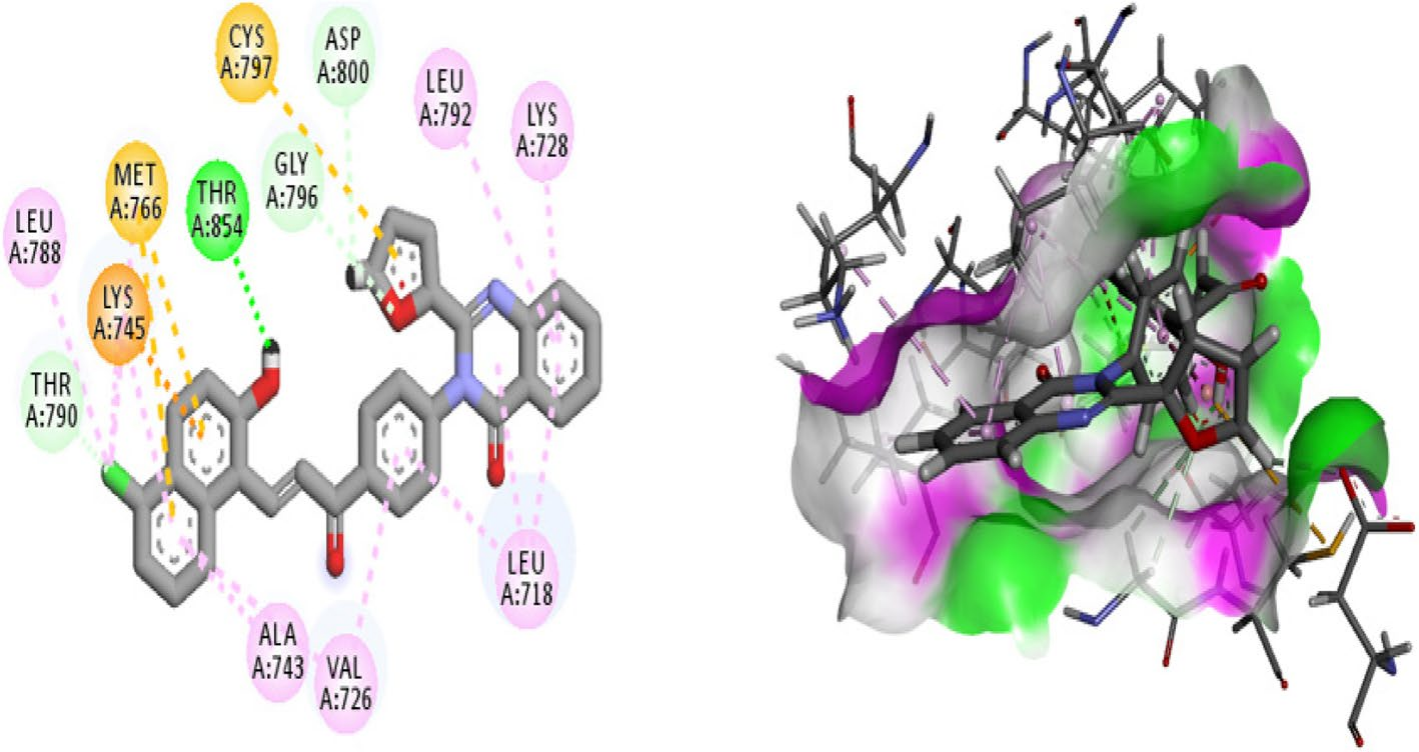
2D and 3D Interactions of designed compound 3 with the active site of the EGFR receptor

Designed compound 3 (Moldock score = -153.96, Rerank score = -105.477) is found to interact with the binding pocket of the EGFR receptor through single conventional hydrogen bonds, four (4) carbon-hydrogen bonds, an electrostatic Pi-cation interaction, three (3) Pi-sulfur interactions, and several alkyl and pi-alkyl hydrophobic interactions. The THR845 residue forms a conventional hydrogen bond with the hydrogen atom of the ortho-hydroxyl group attached to the β-benzene ring. THR790 forms carbon-hydrogen bonds with a chlorine atom attached to the naphthalene group; GLY796 forms two carbon-hydrogen bonds with oxygen and hydrogen atoms of the furyl group; and ASP800 forms the remaining carbon-hydrogen bond with a hydrogen atom of the furyl group. Phenyl rings of the Naphthalene group intercalated in space to form an electrostatic Pi-Cation interaction with LYS745, two (2) Pi-Sulfur interactions with MET766, and CYS797 forms the other Pi-Sulfur interaction with the Furyl ring moiety. LYS745, MET766 and LEU788 form alkyl hydrophobic interactions, while LEU718, LYS728, LEU792, VAL726, ALA743, and LYS745 residues form several pi-alkyl hydrophobic interactions. Fig. 13 shows the 2D and 3D interactions of designed compound 3 in the active site of the EGFR receptor.

**Fig. 14:**
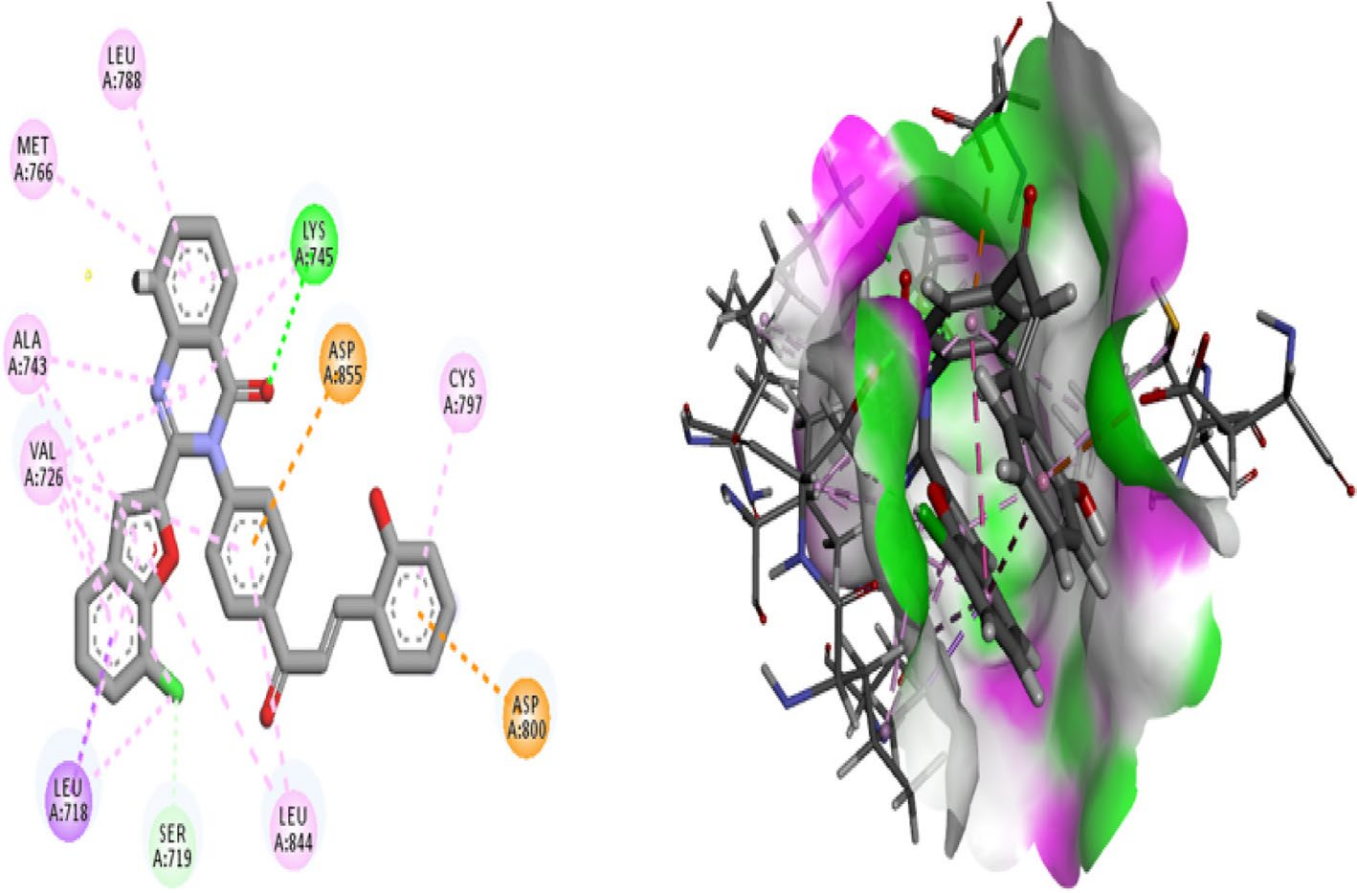
2D and 3D Interactions of designed compound 5 with the active site of the EGFR receptor

Designed compound 5 (MolDock score = -150.758, Rerank score = -84.7712) interacted with the active site of the EGFR receptor via a single conventional and carbon-hydrogen bond, two (2) electrostatic Pi-anion, one hydrophobic Pi-Sigma, and several alkyl and Pi-Alkyl interactions. LYS745 forms a conventional hydrogen bond with the carbonyl oxygen of the quinazoline group. SER719 forms a carbon-hydrogen bond with a chlorine atom attached to the benzofuran group. Benzene rings adjacent to the carbonyl group and the other at the -position are intercalated in space to form electrostatic Pi-anion interactions with ASP855 and ASP800. LEU718 forms a Pi-Sigma hydrophobic interaction with the benzofuran moiety; LEU718 and VAL726 residues form alkyls, while VAL726, ALA743, LYS745, MET766, LEU788, LEU844, CYS797, and LEU718 form hydrophobic Pi-Alkyl interactions. Fig. 14 shows the 2D and 3D interactions of designed compound 5 in the active site of the EGFR receptor.

**Fig. 15:**
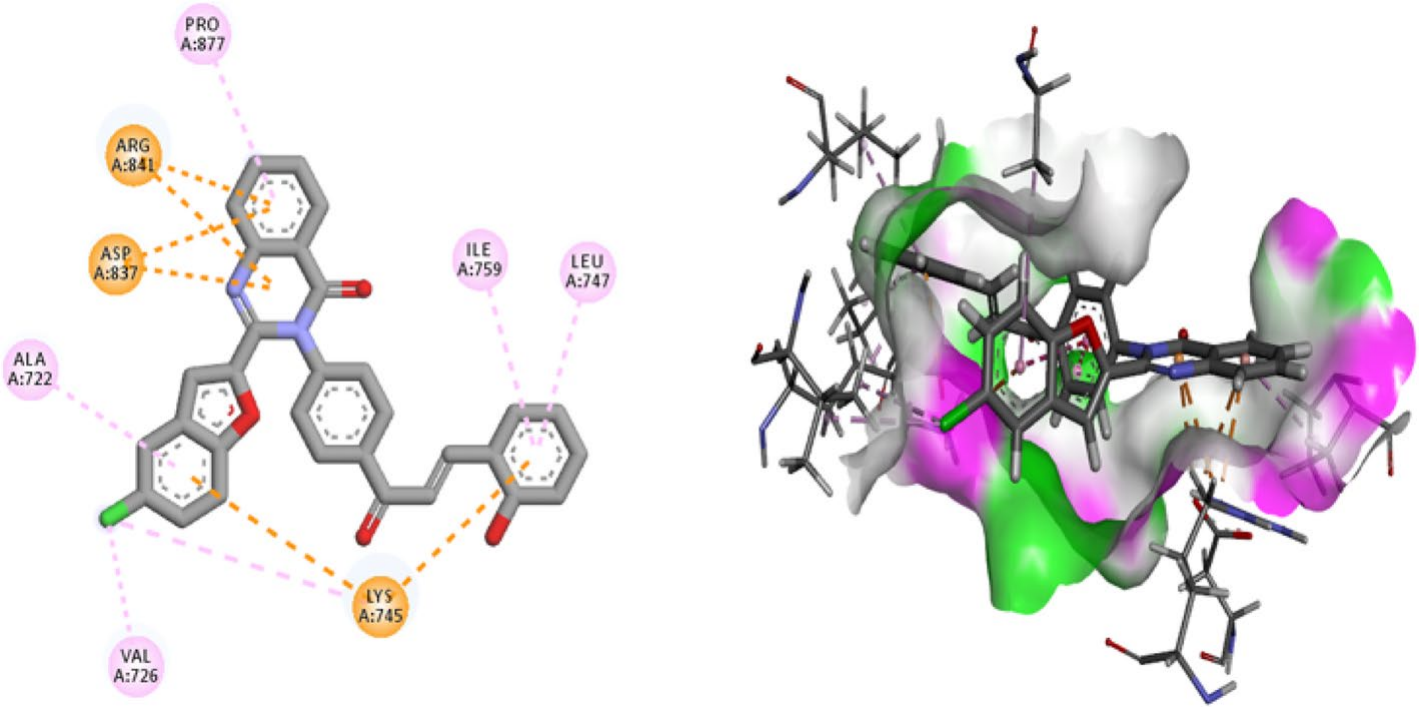
2D and 3D Interactions of designed compound 1 with the active site of the EGFR receptor

Designed compound 1 (MolDock score = -137.652; Rerank score = -100.296) interacted with the EGFR receptor through electrostatic Pi-cations and Pi-anion interactions and hydrophobic alkyl and Pi-alkyl interactions. The benzene rings of the compounds intercalate in space and form electrostatic Pi-cation and Pi-anion interactions with LYS745, ARG841, and ASP837. VAL726 and LYS745 residues form alkyl interactions, while PRO877, LYS745, LEU747, ILE759, and ALA722 form hydrophobic pi-alkyl interactions. Fig. 15 shows the 2D and 3D interactions of design compound 1 in the active site of the EGFR receptor.

This research revealed that Hydrogen bond is the main driving force that regulates the interactions existing between the designed inhibitors and the binding pocket of the EGFR receptor. Low Docking scores exhibited by designed compound 1 is due to the absence of Hydrogen bond interactions between the compound and the protein receptor while higher binding scores of the other designed analogues is due to the presence of several Hydrogen bond interactions between the compounds and the target receptor [13].

Furthermore, to validate the docking studies, the lead compound (5) and the reference drug (DOX) were also docked onto the same binding pocket of the EGFR receptor and it was affirmed that their docking scores is lower than that of the designed compounds. Thus, the designed compounds might serve as novel candidates of EGFR inhibition displaying better capacity than DOX as observed from the docking simulation results.

### Result of Pharmacokinetics and ADMET properties

The results of the pharmacokinetic and ADMET properties studies of the designed quinazoline-4-ones are presented in Tables 7 and 8, respectively. There are high expectations that the designed analogues might possess drug-like properties since they all passed Lipinski’s rule of five (they violate only one of the rules, MW > 500). Synthetic accessibility values of compounds are scaled from 1 (simple to synthesize) to 10 (very difficult to synthesize); their synthetic accessibility values range from 3.74 to 4.06, which suggests that they can be easily synthesized [22]. The designed compounds have a high bioavailability score of 0.55, which indicates that they are well absorbed by the blood plasma. Additionally, the designed compounds displayed high human intestinal absorption, which ranges from 96.265 to 100%, these values exceed the minimal satisfactory absorbance value of 30% [27, 28]. The values of LogBB and LogPS for the designed compounds indicate that they are dispersed uniformly to the brain and are deemed to permeate the central nervous system [24, 25]. Furthermore, they are both substrates and inhibitors of the most crucial class of superenzyme 3A4, which plays a critical role in drug metabolism. A parameter that expresses the linkage between the elimination of a drug per unit time and its amount in the body is the total clearance (TC). These designed inhibitors show reasonable values of TC, which are within the acceptable range of a drug composite in the body. AMES toxicity studies revealed that they are non-toxic [29, 30].

## Conclusions

In this study, coupled GFA-MLR in Material Studio v8.0 was utilized to develop four (4) QSAR models on a series of quinazoline-4-one derivatives. The first model was selected due to its statistical impact with the following parameters: R2 = 0.919, R2adj = 0.898, Ntrain = 25, Q2cv = 0.819, Ntest = 10, and R2pred = 0.7907. The selected model was then employed for the prediction of the pIC­50 of seven (7) newly designed quinazolin-4-one analogues with the ability to inhibit the growth of the MCF-7 breast cancer cell line. The coefficient of determination for the Y-scrambling test (cR2p) performed on the descriptors present in the selected model is found to be 0.7049; this revealed that the model was not obtained by chance correlation and is powerful enough for the prediction of the anti-cancer activities of the compounds. The new novel compounds were designed by using compound 4 as a template due to its high pIC50 value (5.18) and low residual value (-0.01), which are within the defined domain of applicability of the selected model. All the designed compounds have better predicted pIC50 which range from 5.43 to 5.91, compared to the template (5.18) and the reference drug DOX (5.35) used in this study. Moreover, molecular docking studies were done between the designed compounds and the binding pocket of the epidermal growth factor receptor (EGFR) with PDB code 2ITO and were found to have better docking scores than the template and the reference drug. In addition, these drug candidates displayed excellent physicochemical properties. Hence, their synthesis and in vivo and in vitro analyses may affirm the designed analogues as novel EGFR inhibitors for breast cancer treatment.

## Data Availability

Not applicable.

## Abbreviations

QSAR: Quantitative structure activity relationship
EGFR: Epidermal growth factor receptor
DFT: Density functional theory
DOX: Doroxybucin
SDF: Spatial document file
ADMET: Absorption distribution metabolism excretion and toxicity
GFA: Genetic function algorithm
MLR: Multi linear regression
MW: Molecular Weight
HBA: Hydrogen Bond Acceptors
HBD: Hydrogen Bond Donors
TPSA: Topological Polar Surface Area
SA: Synthetic Accessibility
